# Supplementary material for: A Hierarchical Age–Period–Cohort Analysis of Breast Cancer Mortality and Disability Adjusted Life Years (1990–2015) Attributable to Modified Risk Factors among Chinese Women
Source: Int J Environ Res Public Health. 2020 Feb 20;17(4):1367. doi: 10.3390/ijerph17041367 (PMC7068251; doi:10.3390/ijerph17041367)
Supplement: Supplementary file 1 [file ijerph-17-01367-s001.pdf]

# A Hierarchical Age–Period–Cohort Analysis of Breast Cancer Mortality and Disability Adjusted Life Years (1990–2015) Attributable to Modified Risk Factors among Chinese Women

**Sumaira Mubarik, Fang Wang , Saima Shakil Malik , Fang Shi , Yafeng Wang , Nawsherwan and Chuanhua Yu***

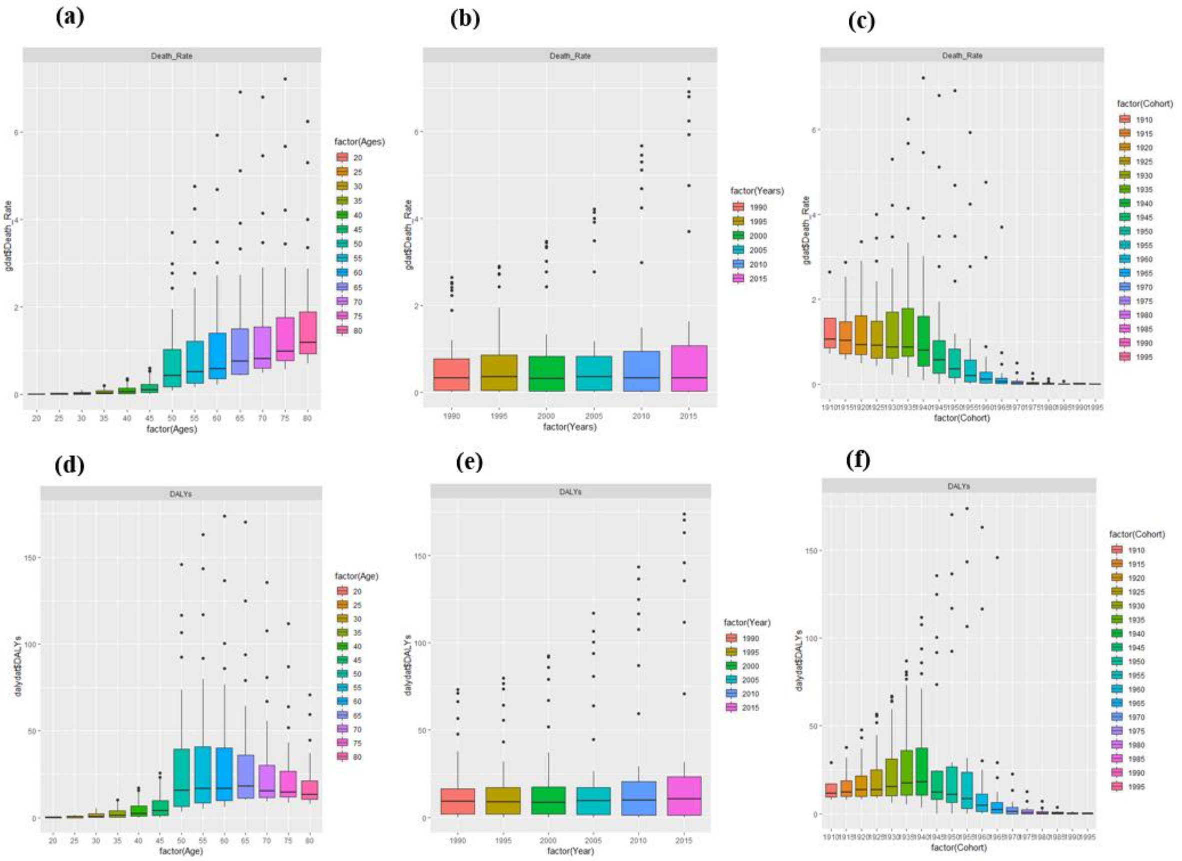


**Figure S1.** Breast cancer Mortality Rates (MRs) and Disability Adjusted Life Years (DALYs) across age (**a,d**), period (years) (**b,e**) and cohorts (**c,f**) attributable to all risk factors together (HBMI, Alcohol use, LowPA and smoking)

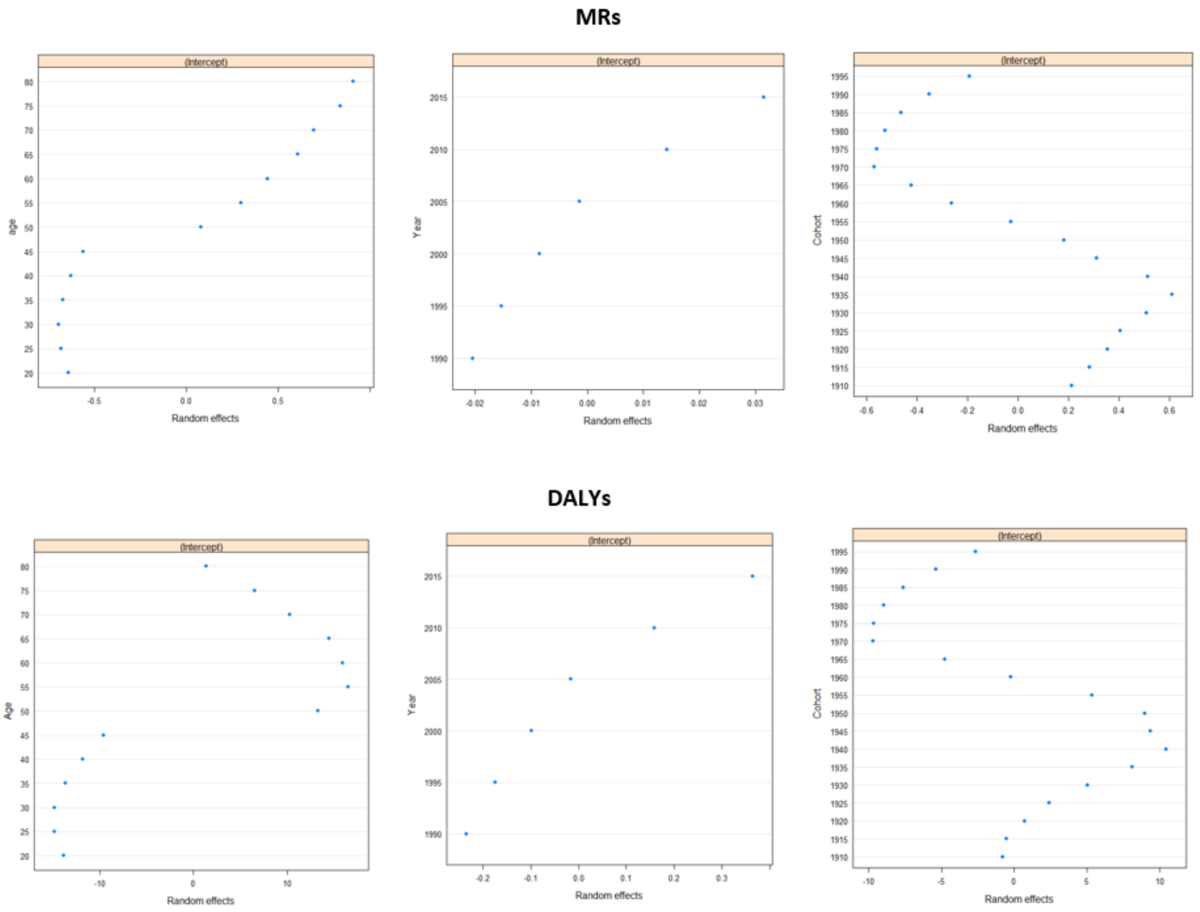


**Figure S2.** Random effects of age, years (period) and cohort on MRs and DALYs from HAPC model for all risk factors (HBMI, Alcohol use, LowPA and smoking)
